# Contemporary Dietary Intake: Too Much Sodium, Not Enough Potassium, yet Sufficient Iodine: The SALMEX Cohort Results

**DOI:** 10.3390/nu10070816

**Published:** 2018-06-25

**Authors:** Olynka Vega-Vega, Jorge I. Fonseca-Correa, Angeles Mendoza-De la Garza, Rodolfo Rincón-Pedrero, Angeles Espinosa-Cuevas, Yolanda Baeza-Arias, Omar Dary, Bertha Herrero-Bervera, Iris Nieves-Anaya, Ricardo Correa-Rotter

**Affiliations:** 1 Nephrology and Mineral Metabolism Department, Instituto Nacional de Ciencias Médicas y Nutrición Salvador Zubirán, Mexico City 14080, Mexico; olynkavega@hotmail.com (O.V.-V.); jorgefonseca84@hotmail.com (J.I.F.-C.); angelesmendozag@gmail.com (A.M.-G.); rinconpedrero@gmail.com (R.R.-P.); angeles.espinosac@incmnsz.mx (A.E.-C.); drayolandabaeza@hotmail.com (Y.B.-A.); herrerobertha@hotmail.com (B.H.-B.); iris.nanaya@hotmail.com (I.N.-A.); 2 Nutrition Division, Bureau for Global Health, US Agency for International Development, Washington, DC 20004-4810, USA; omardaryphn@gmail.com

**Keywords:** salt intake, sodium-potassium ratio, iodine intake

## Abstract

Initiatives to reduce sodium intake are encouraged globally, yet there is concern about compromised iodine intake supplied through salt. The aim of the present study was to determine baseline sodium, potassium, and iodine intake in a sample of workers from our Institution in Mexico City (SALMEX Cohort). **Methods**. From a cohort of 1009 workers, appropriate 24-h urine and three-day dietary recall was collected in a sample of 727 adult subjects for assessment of urinary sodium, potassium, and iodine concentrations. Median urinary iodine excretion (UIE) was compared across categories of sodium intake of <2, 2–3.6, and ≥3.6 g/day. **Results**. Average sodium intake was 3.49 ± 1.38 g/day; higher in men than women (4.14 vs. 3.11 g/day, *p* ≤0.001). Only 10.6% of the population had sodium intake within the recommended range (<2 g/day); 45.4% had high (2–3.6 g/day) and 44% had excessive intake (>3.6 g/day). Average urinary Na/K ratio was 3.15 ± 1.22 (ideal < 1), higher in men (3.42 vs. 3.0, *p* ≤ 0.001). The multivariate analysis showed that sodium intake was associated with age (*p* = 0.03), male sex (*p* < 0.001), caloric intake (*p* = 0.002), UKE (*p* < 0.001) and BMI (*p* < 0.001). Median iodine intake was 286.7 µg/day (IQR 215–370 µg/day). Less than 2% of subjects had iodine intake lower than recommended for adults (95 µg/day); 1.3% of subjects in the recommended range of salt intake had low iodine intake. There is a direct relationship between iodine and sodium urinary excretion (*r* = 0.57, *p* < 0.0001). **Conclusions**. In the studied population, there was an excessive sodium intake and an imbalance between sodium and potassium intake. Only 10.6% of the population had sodium intake within the recommended values, but iodine intake in this group appears to be adequate.

## 1. Introduction

Cardiovascular disease (CVD) is currently the leading cause of death and disability worldwide. A shift in dietary patterns from a reliance on fresh produce to processed foods high in sodium may partially explain the increase in CVD in recent decades [1,2]. High sodium intake increases the risk of CVD and renal disease, stroke, and progression of renal disease [3,4,5,6,7]. Excess sodium intake is indirectly related to obesity and metabolic syndrome through increased thirst and soft drink/sugared beverage consumption [4] and has also been associated with nephrolithiasis, osteoporosis, and higher risk of stomach cancer [8,9,10,11]. Mean sodium intake around the world has been calculated between 2.1–6.6 g/day depending on the country, with a mean around 4 g/day [12]. The increased sodium (Na) intake of the modern western diet is reciprocated by a decrease in dietary potassium (K) intake. Low dietary K may increase the effect of Na on blood pressure (BP), and the relationship between Na and BP becomes stronger if the urinary Na/K ratio is considered [13]. Similarly, low K intake is associated with increased CVD risk [14,15].

Reduction of sodium intake lowers BP in experimental and clinical conditions [16,17]. A meta-analysis of randomized salt reduction trials found that reducing 6 g/day of salt intake may lower systolic/diastolic BP 7/4 mmHg in hypertensive and 4/2 mmHg in normotensive individuals [18]. A population-based modeled projection estimated that reducing salt intake by 3 g/day in the United States would significantly reduce the number of deaths per year from any cause by 44,000 to 92,000. [19]. The World Health Organization (WHO) and Pan American Health Organization (PAHO) have set a target to reduce salt consumption to <5 g/day, or <2 g/day of sodium, in adults by 2020 [20,21]. To reach this goal, an assessment of the current salt intake is required, so the impact of future interventions can be objectively quantitated [22,23].

On the other hand, historical efforts have been made to prevent iodine-deficiency disorders (e.g., cretinism or hypothyroidism), [24] and the WHO has endorsed universal iodization of salt as a vehicle for iodine supplementation [25,26]. Salt iodization was partially implemented in Mexico in 1943 and since 1993 an official regulation states that iodine should be added to salt in a concentration of 30 ± 10 mg/kg [27], applicable to all table salt and salt employed in food processing. Thus, there is concern that iodine deficiency and its consequences may re-emerge if salt intake is reduced. The main aim of this study was to determine the sodium intake in a sample of Mexico City’s population, addressing concomitant potassium as well as iodine intake, in the latter to assess its adequacy when sodium intake is low. We also aimed to determine prevalence of non-communicable diseases (NCD) and their relationship with salt intake. We have previously reported dietary sources of sodium for the adult Mexican population of this cohort [28].

## 2. Materials and Methods 

### 2.1. Study Design and Participants

The Salt and Mexico (SALMEX) cohort study is a cross-sectional, observational, study aimed at assessing the average sodium, potassium, and iodine intake in Mexican population. We included adult men and women who worked at our Institute in Mexico City during data collection period. We excluded subjects with prior diagnosis of congestive heart failure, advanced renal (eGFR <60 mL/min/1.73 m^2^) or hepatic disease, intestinal resection, diuretic initiation in the previous 10 days, active infection, pregnancy, and lactation. 

Primary goals: (1) to determine the average sodium, potassium, and iodine intake stratified by sex; (2) assess prevalence of NCD; and (3) identify eating habits, fluid intake, physical activity, and other risk factors for NCD development. The present refers to results of sodium, potassium, and iodine intake in the cross-sectional evaluation of this cohort at participant inclusion and its association with NCD risk factors. All procedures were conducted following the Declaration of Helsinki and approved by the Ethics Committee of our Institute (reference 09/191). Participation was voluntary, and all subjects gave their informed consent for inclusion before they participated in the study.

### 2.2. Data and Sample Collection

Data was collected between October 2010 and December 2011. Subjects were recruited via informative sessions on the health risk of high sodium diet, delivered to all areas and departments of our Institution. Participants received detailed information, instructions, and material for 24-h urine (24h-U) collection, as well as a standardized questionnaire to assess three-day dietary intake recall. On an appointed date, a medical history, physical exam, and nutritional and body composition evaluation were performed. Laboratory work-up included delivery of the 24h-U collection and a fasting blood sample. 

A standardized WHO-developed questionnaire was applied to participants by a physician (Table S1). Previous known diagnosis and treatment of high BP or diabetes mellitus (DM) were recorded. Three BP measurements were performed on the right arm after a five-minute rest in a sitting position, using an Omron HEM-907XL sphygmomanometer (Omron Health Care Inc., Lake Forest, IL, USA). We added the diagnosis of hypertension (HT) when the mean BP of three resting measurements was ≥140/90 mm/Hg. DM was also diagnosed when fasting plasma glucose (FPG) was ≥126 mg/dL. Body weight and height were measured; subjects were defined as overweight with Body Mass Index (BMI) of 25.0–29.9 kg/m^2^, and obese with ≥30.0 kg/m^2^. 

A three-day diet recall was collected by an experienced dietitian and estimation of the serving size of food portions was aided by standardized plastic/paper models. Data from the survey corresponding to individual food items, macronutrients, and micronutrients were converted to average daily using NutriKcal^®^VO nutrient analysis software (V2) (CONSINFO, S.C., Mexico City, Mexico). More extensive information on nutritional evaluation has been published elsewhere [28]. 

In cases where the 24h-U was clearly inappropriate (urinary tract infection or menses), sample collections were repeated within the next week. Urinary Na (UNa) and K (UK) were quantified with Synchron Cx5 PRO autoanalyser (Beckman Coulter Fullerton, La Habra, CA, USA) (coefficient of variation (CV) = 1.5% for UNa and CV = 1.0% for UK). Urinary creatinine (UCr) was determined according to modified Jaffe method using the same apparatus. Urinary iodine (UI) was determined with a modification of the Sandel–Kolthoff method using ammonium persulfate digestion and microplate reading (405 nm). 

Appropriateness of 24h-U collections was determined by UCr to weight ratio (mg/kg), considering adequate daily UCr excretion as 10–20 mg/kg for women and 15–25 mg/kg for men. Incomplete samples were excluded from the analysis. Urinary sodium, potassium, and iodine excretion rates (UNaE, UKE, and UIE, respectively) were calculated according to each electrolyte’s concentration and the total urinary volume. UIE was calculated dividing the total amount of iodine in the 24h-U by 0.92, assuming that 92% of the total intake is excreted through urine. No adjustment was possible for extra-renal potassium excretion, given that there is no standardized factor to do so.

### 2.3. Statistical Analysis 

The sample size was determined considering the following assumptions: (1) Na intake is different between men and women; (2) WHO considers that samples of 200 people (100 per sex) are sufficient to characterize the group mean with 95% confidence intervals of ±12 mmol/day (±276 mg/day), assuming a standard deviation of UNaE of about 60 mmol/day [29]. Considering a loss of 20% due to inappropriate 24h-U samples, we required a minimum of 240 (120 per sex). In order to reduce the 95% confidence interval (CI) to ±10 mmol/day we determined a sample size of 800 individuals.

Mean, standard deviation, and ranges as well as median and interquartile range (p25–p75) were calculated for all variables and Kolmogorov–Smirnov test used to assess for normality. The association between sex and the studied variables was assessed; for quantitative variables, Student´s *t* test (or Mann–Whitney if the distribution of results was not homogeneous), and for qualitative variables, a χ^2^ test were used. ANOVA or Kruskal–Wallis tests were used to compare more than three groups. Multivariate analysis through linear regression was used to determine association between salt intake and age, sex, HT, DM, BMI, energy intake, UKE. A non-parametric Spearman correlation test was used to assess the relationship between average UNaE and UIE, and the relationship between dietary habits and UNaE. Calculations were made using SPSS software (version 15) (SPSS Inc., Chicago, IL, USA). Statistical significance was set at *p* < 0.05. Graphs were created with GraphPad Prism software.

## 3. Results

We included 1009 subjects, of whom 760 provided an appropriate 24h-U collection and 727 had a full questionnaire available. Table 1 shows general characteristics of study participants: mean age 39.3 years, 11.7% known to have DM and under some form of treatment, 14.5% had a previous diagnosis of HT, and 27% were obese. Considering FPG and BP the day of the interview, HT diagnosis increased to 20.8% (significantly higher in men, 28.8% vs. 16.3%) and DM diagnosis to 12.4% (Table 1).

**Figure 1:**
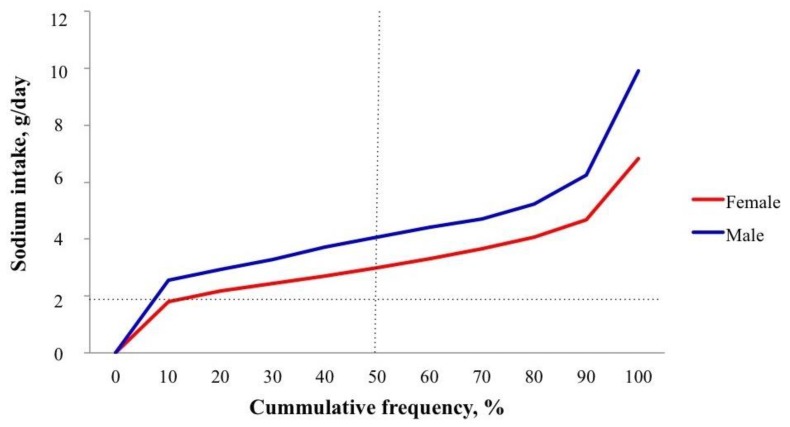
Deciles of sodium intake. The percentage of subjects with sodium intake ≤2 g/day for men 3.8% and women 14.3%, median sodium intake for men (4.2 g/day) and women (3.0 g/day).

Table 2 describes sodium, potassium and iodine excretions between sexes, adjusted and unadjusted to caloric intake. UNaE was significantly higher in men than in women (mean 179.9 vs. 135.3 mmol/day, 4.14 vs. 3.11 g/day; *p* < 0.0001). Only 3.8% of men and 14.3% of women had sodium intake <2 g/day (Figure 1; Table 3). When salt intake was adjusted for caloric intake (kcal/day), levels were similar for both sexes (mean 4.43 mg/kcal in men vs. 4.56 in women, *p* = 0.186; Table 2). The multivariate analysis showed that salt intake was associated with age (*p* = 0.03), male sex (*p* = 0.03), caloric intake (*p* = 0.002), UKE (*p* < 0.001) and BMI (*p* < 0.001), with no association with HT or DM diagnosis, or even Systolic or Diastolic BP (*p* = 0.4, 0.4, 0.05, and 0.6, respectively).

Mean potassium intake was 50.81 mmol/day and was also higher in men than in women (55.62 vs. 48.14 mmol/day; *p* < 0.0001). The mean Na/K ratio was 3.15, and was also higher in men than in women (3.42 vs. 3.01, *p* < 0.0001). When adjusted for caloric intake, the mean K intake was nominally higher in women than in men (0.026 vs. 0.024 mmol/kcal, *p* = 0.004). There were no significant differences in Na/K ratio between subjects with HT or DM as compared to healthy subjects (3.1 vs. 3.2 mmol/day for HT, *p* = 0.12; 3.0 vs. 3.1 mmol/day for DM, *p* = 0.34)*.*

We divided the sample into three groups according to sodium intake: Recommended (<2 g/day of sodium), high (2–3.6 g/day of sodium) and very high salt intake (>3.6 g/day of sodium) (Table 3). Only 10.6% of the population had sodium intake values within the recommended range; 45.4% had high values, and 44% had very high values. When analyzed by sex, males had higher prevalence of “excessive” (group 3) sodium intake (63.9% vs. 33%, *p* < 0.001) and lower “recommended” sodium intake (3.8% vs. 14.3%, *p* < 0.001) Higher sodium intake in females was associated with higher BP (even within “normal” range), HT diagnosis, weight, BMI, and caloric intake. In males, this association persisted for Systolic BP, HT diagnosis, weight, BMI but not caloric intake. This could be explained by the small number of males in group 1 (“recommended”) that could bias the result. 

**Figure 2:**
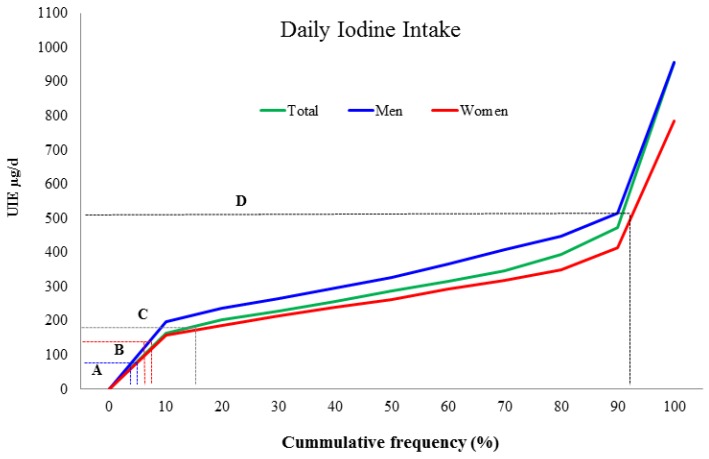
Distribution of iodine intake according to urinary iodine excretion. The green line represents the total sample, men are represented in blue and women in red. A. Blue dotted lines represent the estimated average requirement of iodine (EAR = 95 µg/day); only 8 women and one man had less than this value. B. Red dotted lines represent the recommended daily allowance of iodine (RDA = 150 µg/day); 70 subjects (9.6%) had less than this value. C. Gray dotted lines represent the minimum intake allowance for adult women (180 µg/day); 101 women (14%) had less than this value. D. Black lines represent the maximum intake recommended for adult women (500 µg/day); only 17 women (2.3%) had more than this value. There were no subjects with UIE >1000 µg/day.

The median UIE was higher in men than in woman (327.3 vs. 261.2 µg/day; *p* < 0.0001), following a similar pattern as salt intake. When adjusted for caloric intake, UIE was nominally similar for both sexes (Table 2). Only 1.8% of the total population had an UIE value that suggests an iodine intake lower than the estimated average requirement (<95 µg/day), and 8.1% consumed more than the tolerable upper intake level for adult women (>500 µg/day) (Figure 2). Iodine intake is directly proportional to Na intake when both are adjusted to caloric intake (*r* = 0.57, *p* < 0.000). 

When analyzing mean sodium intake according to BMI, HT or DM status, obese subjects showed higher mean intake than overweight and normal BMI subjects (3.99 vs. 3.55 vs. 2.98 g/day respectively; *p* < 0.0001). Hypertensive and diabetic subjects had higher mean salt intake than normotensive and non-diabetic subjects (3.92 vs. 3.37 g/day for HT, *p* < 0.001; 3.8 vs. 3.4 g/day for DM, *p* = 0.01) (HT and DM status were defined considering previous and incidental diagnosis upon evaluation). Educational level, as well as perception of salt intake and eating habits (i.e., days eating out, adding salt at the table) did not influence salt intake. Notably, subjects with self-perceived low salt intake actually consumed a high amount of sodium (2.5 ± 1.4 g/day of salt, i.e., ≥2 g/day).

Estimation of sodium intake through three-day dietary recall questionnaire correlated poorly with the UNaE measurement (gold standard) (r = 0.2, *p* < 0.001). Mean measured sodium intake was on average 2.7 ± 0.12 g/day higher than estimated through questionnaire. Mean daily caloric intake was in average 400 kcal more in men than in women (1976.44 ± 173.74 vs. 1570.75 ± 121.21, *p* = 0.004; Table 1). There was a linear, yet small, correlation between caloric intake and salt consumption per day: for every 500 kcal of energy consumed, subjects consumed 2.11 g of salt 0.83 sodium (r = 0.36, *p* = 0.0001). 

## 4. Discussion

Most participants in this study had an excessive sodium consumption; almost 90% of the population had an intake higher than the WHO-recommended daily salt intake. Interestingly, 83% of them (*n* = 603) perceive themselves as eating within the recommended salt intake (Table 3). In a sub-analysis of this study [28] focused on dietary sources of sodium intake, we previously reported a preliminary overall mean sodium intake of 3.4 ± 1.39 g/day (8.74 ± 3.4 g/day of salt), with the same disparities when comparing 24h-U estimations with dietary recall. The only previous report on salt consumption in the Valley of Mexico City comes from a small study performed in 1996, which included 155 adult individuals. They described a substantially lower salt consumption than our results (2.75 g/day of sodium) [30]. This difference could be explained by a dietary evolution over more than 20 years; present diet is more abundant in industrialized products as compared to what was eaten two decades ago. Furthermore, and most importantly, along the last couple of decades, obesity and overweight have increased systematically in Mexico. A higher caloric consumption is a key element to explain the high prevalence of overweight (40%), and obesity (27%) in our population and contributes strongly to the increased incidence of NCD [31]. As demonstrated in our study, obesity and a high caloric intake correlate with an increased sodium intake. The caloric intake estimations performed in this study are lower than expected, especially considering the prevalence of overweight and obesity. This suggests that the self-report of consumed foods underestimates the actual intake. As in other studies [32], we found a very poor correlation between sodium intake estimated by the three-day dietary intake diaries and the one calculated from UNaE measurement. 

In addition to the excess–sodium intake, K intake was less than half the daily recommendation (120 mmol/day) [33]. This translates to a high Na/K ratio, typical of the modern western diet. Also, BP was higher with higher sodium intake and subjects with HT had a higher sodium intake and Na/K ratio. These data suggest that guideline recommendations or consensus opinions of Na reduction and K increase are not being achieved in the Mexican population. 

Although UNaE level is a close reflection of Na intake, 30% of total K intake is excreted via the gastro-intestinal tract [34]. Consequently, we underestimate K but not Na intake from urinary excretion rates. Nevertheless, assuming that fecal K excretion levels were one third of the total intake, adding this amount to the UKE of each individual would still result in a lower mean K intake than the daily recommendation. Unlike iodine, there is no numerical factor that helps correct for this underestimation, due to individual variability. 

One notable finding in our study regards iodine consumption. In 2013, the Federal Commission for the Protection Against Sanitary Risk (COFEPRIS) estimated that the national average content of iodine in salt in Mexico was 30 mg/kg. Given that the primary vehicle for iodine fortification is salt, there is concern that decreasing salt consumption will increase the risk of iodine deficiency and it is recommended to adjust adequate iodine supplementation to reduced salt intake [35]. Subjects in this study have in most instances an optimal iodine intake status, as only 1.3% of individuals had an estimated iodine intake below 95 μg/day—the estimated average requirement- and 5.1% below 150 μg/day—the Recommended Daily Intake. This shows that the current salt iodization program of Mexico is providing enough iodine to the population according to the current salt intakes. More importantly, we demonstrate here for the first time that iodine status is not compromised in the great majority of Mexican adults from this sample who had sodium intake in line with the WHO recommendations of <2 g/day. If all of the studied population consumed the recommended amount of salt, <3% would have UIE below the estimated average requirement (95 µg/day). The risk-benefit balance should therefore continue to lean toward lower salt intake over risk of iodine deficiency. 

The strength of our methodology is the use of 24h-U collection for the assessment of usual sodium, potassium, and iodine intake. Limitations to the study include that we enrolled a sample of the total population of workers from our Institution (approximately 30%) which is located in the South part of Mexico City, and that, given that it is a National Health Institution, the educational level of the subjects enrolled is higher than the general population. However, we observed no differences in salt intake due to educational level within the cohort. An additional limitation is the small size of the subgroup of subjects with salt intake <5 g/day (*n* = 77); although it gives strength to the concept that only a small proportion of the population consumes the recommended sodium intake, it limits the generalizability of other conclusions, for example, the appropriateness of iodine intake in spite of low sodium intake. 

## 5. Conclusions

In conclusion, our findings indicate that sodium intake in our population is higher than recommended, but adults who have UNaE values equivalent to sodium intakes of <2 g/day do not have a compromised iodine intake. Thus, programs focused on reducing sodium intake to prevent hypertension could proceed without concern of proper iodine supplementation. Nevertheless, frequent national monitoring and surveillance will be required to assess the impact of changes to the food’s sodium content and the contribution of iodized salt from such processed foods. Additionally, our study confirms an expected positive correlation between Na intake and hypertension. We also demonstrate that males (vs. females) and obese subjects had a higher sodium intake than overweight and normal BMI individuals, highlighting the relationship between caloric and sodium intake. These results confirm the need of a fully integrated educational approach to the current western alimentary behavior, which should include calorie reduction, sodium intake reduction, and potassium intake increase.

## Figures and Tables

**Table 1:** Characteristics of the study population by sex.

Variable	Total *n* = 727	Men *n* = 260	Women *n* = 467	*p* *
Age, years	39.31 ± 10.57	38.23 ± 10.71	39.93 ± 10.45	0.04
Present smokers, %	24.9	37.3	18.0	<0.001
College educated, %	45.8	38.5	49.9	0.003
HT, %	20.8	28.8	16.3	<.001
DM, %	12.4	10.8	13.3	0.32
Weight, kg	70.9 ± 14.6	79.6 ± 14.4	66.1 ± 12.3	<0.001
Height, m	1.6 ± 0.09	1.69 ± 0.07	1.55 ± 0.06	<0.001
BMI, kg/m^2^	27.5 ± 4.68	27.8 ± 4.35	27.4 ± 4.8	0.40
Overweight, %	41.1	44.2	39.4	0.27
Obese, %	27.3	28.1	27.0	0.73
Systolic BP, mmHg	120.30 ± 15.12	127.30 ± 74.16	116.41 ± 13.80	<0.001
Diastolic BP, mmHg	75.86 ± 9.72	78.93 ± 10.19	74.16 ± 9.03	<0.001
eGFR (CKD-EPI) mL/min/1.73 m^2^	107.0 ± 14.70	104.77 ± 14.37	108.25 ± 14.75	0.02
Energy intake, kcal/day	1709.42 ± 244.85	1976.44 ± 173.74	1560.75 ± 121.2	0.004

* Comparison between sexes. Values are mean ± SD or percentages. BMI, body mass index; BP, Blood pressure; DM, Diabetes mellitus; eGFR, estimated Glomerular Filtration Rate; HT, hypertension; SD, standard deviation. Definitions of HT and DM are described in Methods.

**Table 2:** Twenty-four-hour urine collection data.

Variable	Total *n* = 727	Men *n* = 260	Women *n* = 467	*p* *
Volume, mL	1613.5 ± 767.5	1657.5 ± 782.0	1589.1 ± 759.1	0.252
Na, mmol/day	151.32 ± 60.2	179.9 ± 65.6	135.3 ± 50.4	<0.001
Na (energy adjusted), mmol/kcal/day	0.075 ± 0.03	0.077 ± 0.037	0.074 ± 0.32	0.186
K, mmol/day	50.81 ± 18.1	55.62 ± 18.95	48.14 ± 17.07	<0.001
K, mmol/kcal/day	0.025 ± 0.011	0.024 ± 0.011	0.026 ± 0.11	0.004
Urinary Na/K ratio	3.15 ± 1.22	3.42 ± 1.22	3.01 ± 1.19	<0.001
Na intake (24-hr urine), g/day	3.49 ± 1.38	4.14 ± 1.51	3.11 ± 1.16	<0.001
Na intake (quiestionnaire), g/day	2.6 ± 0.98	2.98 ± 1.16	2.38 ± 0.79	<0.001
Na intake (energy adjusted, 24-h urine), mg/kcal/day	1.73 ± 0.8	1.82 ± 0.87	1.7 ± 0.75	0.186
Iodine intake, µg/day **	286.7 (215.2–370.3)	327.3 (250.9–430.9)	261.2 (201.5–334.7)	<0.001
Iodine intake (energy adjusted), µg/kcal/day **	0.16 (0.12–0.21)	0.17 (0.13–0.22)	0.17 (0.13–0.21)	<0.001

* Comparison between sexes. Values are mean ± SD except for iodine intake. ** Iodine intake = Iodine excretion/0.92, K = potassium, Na = sodium; IQR = interquartile range; SD = standard deviation.

**Table 3:** Distribution according to sodium intake groups.

	***Overall (n = 727)***				***Female (n = 467)***				***Male (n = 260)***			
	**Group 1 ^(a) ^** ***n* = 77** **(10.6%)**	**Group 2** ***n* = 330** **(45.4%)**	**Group 3** ***n* = 320 (44.0%)**	***p***	**Group 1** ***n* = 67** **(14.3%)**	**Group 2** ***n* = 246 (52.7%)**	**Group 3** ***n* = 154 (33.0%)**	***p***	**Group 1** ***n* = 10** **(3.8 %)**	**Group 2** ***n* = 84** **(32.3%)**	**Group 3** ***n* = 166** **(63.9%)**	***p***
Age, year *	38.2 ± 10.6	40.1 ±11.1	38.8 ± 9.7	0.19	38.5 ±11.0	40.7±11.0	39.4±9.3	0.23	36.3±8.5	38.4±12.3	38.3±9.9	0.84
Perception of salt intake ^(b)^^†^	603 (83)	119 (16.4)	4 (0.6)	-	384 (82.2)	80 (17.1)	3 (0.6)	-	220 (84.2)	39 (15)	1 (0.4)	0.4 ^(c)^
Na intake, g/day *	1.6 ± 0.3	2.7 ± 0.4	4.7 ± 1.1	<0.001	1.6 ± 0.3	2.7 ± 0.4	4.4 ± 0.8	<0.001	1.7 ± 0.3	2.8 ± 0.4	4.9 ± 1.3	<0.001
Urinary Na/K ratio *	2.1 ± 0.9	2.9 ± 1.0	3.7 ± 1.2	<0.001	2.1 ± 0.9	2.8 ± 1.0	3.7 ± 1.2	<0.001	1.9 ± 0.7	3.0 ± 1.0	3.7 ± 1.2	<0.001
SBP, mmHg * DBP, mmHg *	113.7 ± 11.9 72.7 ± 8.4	118.4 ± 14.2 74.8 ± 9.0	123.8 ± 15.9 75.9 ± 10.4	<0.001 <0.001	111.9 ± 10.3 72.0 ± 7.8	116.4 ± 13.5 74.0 ± 8.6	118.4 ± 15.2 74.2 ± 9.0	0.005 0.03	126.3 ± 15.0 76.9 ± 11.4	124.3 ± 14.8 77.0 ± 9.8	128.9 ± 14.8 80.0 ± 10.2	0.05 0.07
Weight, kg *	62.2 ± 11.6	67.7 ± 12.4	76.9 ± 15.1	<0.001	59.7 ± 8.4	64.9 ± 12.1	70.9 ± 12.4	<0.001	79.3 ± 15.7	74.1 ± 10.4	82.5 ± 15.3	<0.001
BMI, kg/m^2 ^*	25.3 ± 3.8	26.8 ± 4.5	28.8 ± 4.7	<0.001	25.0 ± 3.7	27.1 ± 4.7	29.0 ± 5.0	<0.001	27.0 ± 4.7	26.2 ± 3.6	28.7 ± 4.5	<0.001
Hypertension^†^	8 (10.4)	59 (17.9)	84 (26.3)	0.002	4 (6.0)	45 (18.3)	27 (17.5)	0.04	4 (40)	14 (16.7)	57 (34.3)	0.01
Diabetes Mellitus ^†^	7 (9.1)	41 (12.4)	42 (13.1)	0.62	5 (7.5%)	32 (13.0%)	25 (16.2%)	0.2	2 (20)	9 (10.7)	17 (10.2)	0.62
eGFR ^(d)^ *, mL/min/1.73 m^2^	108.4 ± 14.0	105.1 ±15.0	107.7 ± 14.6	0.26	108.9 ± 14.5	107.3 ± 14.7	109.5 ± 14.8	0.33	104.8 ± 9.3	102.4 ± 15.2	106.0 ± 14.1	0.17
Energy intake, kcal/day *	1606.90 ± 201.80	1654.40 ± 222.50	1790.82 ± 251.72	<0.001	1544.78 ± 99.38	1550.96 ± 121.24	1583 ± 10.24	0.01	2023 ± 224	1957 ± 168	1983 ± 173	0.37
UIE, µg/day ^	196.9 (163.4–265.3)	273.54 (221.0–347.4)	371.3 (306.0–4770)	<0.001	195.22 (159.4–263.6)	268.0 (217.4–343.5)	342.6 (277.3–434.5)	<0.001	235.5 (223.6–305.2)	283.4 (234.7–383.2)	405.3 (323.1–504.0)	<0.001
**Iodine Intake Categories (according to UIE)**	***Overall***				***Female***				***Male***			
	**Group 1** ^(a)^ ***n* = 76**	**Group 2** ***n* = 329**	**Group 3 *n* = 316**	**Total** ***n* = 721**	**Group 1** ***n* = 67**	**Group 2** ***n* = 245**	**Group 3** ***n* = 151**	**Total** ***n* = 463**	**Group 1** ***n* = 9**	**Group 2** ***n* = 84**	**Group 3** ***n* = 165**	**Total** ***n* = 258**
<95 µg/day (less than EAR) ^†^	1 (1.3)	5 (1.5)	1 (0.3)	7 (1.0)	1 (1.5)	4 (1.6)	1 (0.7)	6 (1.3)	0 (0)	1 (1.2)	0 (0)	1 (0.4)
95–150 µg/day (RDA) ^†^	18 (23.7)	17 (5.2)	2 (0.6)	37 (5.1)	17 (25.4)	11 (4.5)	1 (0.7)	29 (6.3)	1 (11.1)	6 (7.1)	1 (0.6)	8 (3.1)
150–180 µg/day ^†^	17 (22.4)	33 (10.0)	7 (2.2)	57 (7.9)	16 (23.9)	26 (10.6)	4 (2.6)	46 (9.9)	1 (11.1)	7 (8.3)	3 (1.8)	11 (4.3)
181–500 µg/day ^†^	36 (47.4)	265 (80.5)	260 (82.3)	561 (77.8)	30 (44.8)	199 (81.2)	127 (84.1)	356 (76.9)	6 (66.7)	66 (78.6)	133 (80.6)	205 (79.5)
>500 µg/day ^†^	4 (5.3)	9 (2.7)	46 (14.6)	59 (8.2)	3 (4.5)	5 (2.0)	18 (11.9)	26 (5.6)	1 (11.1)	4 (4.8)	28 (17.0)	33 (12.8)

^(a) ^Group definition: Group (1) Recommended sodium intake, <2 g/day (<5 g/day of salt); (2) High sodium intake 2–3.6 g/day (5–9 g/day of salt); (3) Excessive sodium intake <3.6 g/day (>9 g/day of salt). ^(b)^ Perception of salt intake through question: “In what group do you think you belong: recommended, high, or excessive intake?”. ^(c)^ Comparison of proportions between sexes. ^(d)^ eGFR calculated with CKD-EPI formula. * Mean ± SD; ^ median (Interquartile range); ^†^ *n* (%). BMI, body mass index; DM, diabetes mellitus diagnosis; DPB, diastolic blood pressure; eGFR, estimated glomerular filtration rate; HT, hypertension diagnosis; SBP, systolic blood pressure; UIE, urinary iodine excretion; EAR, estimated average requirement; RDA, recommended daily allowance; UIE, urinary iodine excretion.

## Supplementary Materials

The following are available online at http://www.mdpi.com/2072-6643/10/7/816/s1, Table S1: SALMEX Study sodium intake detection questionnaire (Translated from Spanish).
