# Supplementary material for: Contemporary Dietary Intake: Too Much Sodium, Not Enough Potassium, yet Sufficient Iodine: The SALMEX Cohort Results
Source: Nutrients. 2018 Jun 25;10(7):816. doi: 10.3390/nu10070816 (PMC6073158; doi:10.3390/nu10070816)
Supplement: Supplementary file 1 [file nutrients-10-00816-s001.pdf]

**Table S1**: SALMEX Study sodium intake detection questionnaire (Translated from Spanish).

| **Information** | | |
|---|---|---|
| **Date and origin** | | **Answer** |
| 1 | Delegation or municipality | |
| 2 | Interviewer ID | |
| 3 | Date | └─┴─┘ └─┴─┘ └─┴─┴─┴─┘<br>Day Month Year |

| **Infomed Consent and Name** | | **Answer** |
|---|---|---|
| 4 | Informed consent was read | Yes (1) □<br>No (0) □ **If answer is NO**, read informed consent |
| 5 | Language | Spanish X |
| 6 | Time of interview | Hour: minute └─┴─┘ : └─┴─┘ |
| 7 | Last name | |
| 8 | First name(s) | |
| **Aditional information** | | |
| 9 | Telephone number (when available) | |

| **DEMOGRAPHIC INFORMATION** | | |
|---|---|---|
| **Question** | | **Answer** |
| 10 | Sex | Male (1) □<br>Female (0) □ |
| 11 | Place of birth<br>*If unknown, leave blank* | └─┴─┘ └─┴─┘ └─┴─┴─┴─┘<br>Day Month Year |
| 12 | What is your highest educational level? | No formal schooling (1) □<br>Elementary school (2) □<br>Middle school (3) □<br>Highschool (4) □<br>Undergraduate studies (college or university) (5) □<br>Graduate studies (6) □<br>Refuses to answer (7) □ |
| 13 | What is your marital status? | Never married (1) □<br>Married (2) □<br>Separated (3) □<br>Divorced (4) □<br>Widowed (5) □<br>Living in union (6) □<br>RJefuses to answer (88) □ |
| 14 | Describe your main job in this institution for the last 12 months. | Department ________________<br>Position ________________ |

| | | |
|---|---|---|
| | | |

| TOBACCO CONSUMPTION | | |
|---|---|---|
| **Question** | | **Answer** |
| 15 | Do you currently smoke any tobacco related product? | Yes □ (1) No □ (0) |
| 16 | Do you currently smoke daily? | Yes □ (1) No □ (0) |
| 17 | At what age did you start smoking? | Age (years) ⌊_⌊_⌋<br>Doesn't know □ |
| 18 | In average, how many of these tobacco products do you smoke daily? | Cigarettes ⌊_⌊_⌋<br>Cigars ⌊_⌊_⌋ |
| 19 | In the past, did you ever smoke daily? | Yes □ (1) No □ (0) |
| 20 | At what age did you stop smoking daily? | Age (años) ⌊_⌊_⌋<br>Doesn't know □ |

| ALCOHOL CONSUMPTION | | |
|---|---|---|
| **Question** | | **Answer** |
| 21 | Have you ever consumed any alcoholic beverage? | Yes □ (1) No □ (0)<br>*If NO, go te next sextion (DIET)* |
| 22 | Have you consumed any alcoholic beverage in the last 12 months? | Yes □ (1)<br>No □ (0) |
| 23 | During the last 12 months, how frequently did you consume any alcoholic beverage? | Daily □ (1)<br>5-6 days a week □ (2)<br>1-4 days a week □ (3)<br>1-3 days a month □ (4)<br>Less than once a month □ (5)<br>Never □ (6) |
| 24 | Have you consumed any alcoholic beverage in the last 30 days? | Yes □ (1)<br>No □ (0)<br>*If NO, go to next section (DIET)* |
| 25 | During the last 30 days, hay many times did you consume at least one alcoholic beverage? | Number of Days ⌊_⌊_⌋<br>Doesn't know □ |
| 26 | During the last 30 days, when consuming alcoholic beverages, how many drinks did you consume in average per day? | Number of beverages ⌊_⌊_⌋<br>Doesn't know □ |
| 27 | During the last 30 days, what was the highest number of drinks you had in one day, including any type of alcoholic beverage? | Number of beverages ⌊_⌊_⌋<br>Doesn't know □ |

| | | |
|---|---|---|
| 28 | During the last 30 days, how many times did you consume five or more drinks (men) or four or more drinks (women)? | Number of days ⌊_⌊_⌋<br>Doesn't know □ |

| | | |
|---|---|---|
| 29 | During the last 30 days, when consuming an alcoholic beverage, how many times did you do it with food? (do not include snacks) | Usually with food □ (1)<br>Sometimes with food □ (2)<br>Rarely with food □ (3)<br>Never with food □ (4)<br>Hasn't consumed alcohol □ (5) |
| 30 | During the last 7 days, how many drinks of any alcoholic beverage did you have for any given day? | Monday \|_\|_\|<br>Tuesday \|_\|_\|<br>Wednesday \|_\|_\|<br>Thursday \|_\|_\|<br>Friday \|_\|_\|<br>Saturday \|_\|_\|<br>Sunday \|_\|_\| |

| DIET | | |
|---|---|---|
| **Question** | | **Answer** |
| 31 | During a typical week, how many days do you eat fruit? | Number of days \|_\|_\|<br>Doesn't know □ |
| 32 | How many portions of fruit do you eat for each day? | Number of portions \|_\|_\|<br>Doesn't know |
| 33 | During a typical week, how many days do you eat vegetables? | Number of days \|_\|_\|<br>Doesn't know □ |
| 34 | How many portions of vegetables do you eat for each day? | Number of portions \|_\|_\|<br>Doesn't know □ |
| 35 | What kind of oil or fat do you use to prepare food? | Vegetable oil□ (1)<br>Lard □ (2)<br>Butter □ (3)<br>Margarine □ (4) |

| PHYSICAL ACTIVITY | |
|---|---|
| **Question** | **Answer** |

| During job hours | | |
|---|---|---|
| 45 | Does your job imply carrying out intense physical activity that accelerates your breathing or heart rate (such as lifting weight, shoveling, construction) for at least 10 minutes? | Yes□ (1)<br>No □ (0) |
| 46 | During a typical work week, how many days do you have to carry out intense physical activity during work? | Number of days |
| 47 | During one of these dayse, how much time do you spend doing intense physical activity? | Hours: Minutes: |
| 48 | Does your job imply carrying out moderate physical activity that slightly accelerates your breathing or heart rate (such as walking fast or carrying light weights) for at least 10 minutes? | Yes□ (1)<br>No □ (0) |
| 49 | During a typical work week, how many days do you have to carry out moderate physical activity during work? | Number of days |
| 50 | During one of these dayse, how much time do you spend doing moderate physical activity? | Hours: Minutes: |
| **Transportation to your job** | | |
| 51 | Do you walk or use bicycle for at least 10 straight minutes to get to work? | Yes□ (1)<br>No □ (0) |
| 52 | How many days a week do you walk or use bicycle for at least 10 straight minutes to get to work? | Number of days |
| 53 | How much time do you spend walking or using bicycle for at least 10 straight minutes to get to work? | Hours: minutes: |

| | | |
|---|---|---|
| | | None in particular (6)<br>Doesn't use □ (7)<br>Doesn't know □ (8) |
| **Knowledge, attitude, behaviour towards salt in diet.** | | |
| **Question** | | **Anser** |
| 36 | In a typical week, how many days do you eat out of home? | Number of days<br>Doesn't know |
| 37 | Do you add salt to your food at the table?<br>*(Choose one)* | Never □ (0)<br>Rarely □ (1)<br>Occasionally □ (2)<br>Usually □ (3)<br>Always □ (4) |
| 38 | Do you add salt to your food when cooking?<br>*(Choose one)* | Never □ (0)<br>Rarely □ (1)<br>[illegible]<br>Usually □ (3)<br>Always □ (4) |
| 39 | How much salt do you think you consume?<br>*(Choose one)* | Excessive□ (1)<br>High □ (2)<br>Recommended □ (3)<br>Little □ (4)<br>Very little □ (5)<br>Doesn't know □ (6)<br>Refuses □ (88) |
| 40 | Do you think a high salt diet can cause health problems? | Yes □ (1)<br>No □ (0)<br>Doesn't know □ (3)<br>Refuses □ (88) |
| 41 | What kind of health problems do you think a high-salt diet can cause?<br>*(Choose one or more)* | High blood pressure□ (1)<br>Osteoporosis□ (2)<br>Stomach cancer□ (3)<br>Kidney stones□ (4)<br>None of the above □ (5)<br>All of the above □ (6)<br>Doesn't know □ (7)<br>Refuses □ (88) |
| 42 | How important is salt/sodium in your diet? | Not important □ |

| | | |
|---|---|---|
| | | Of Little importance □<br>Very important □ |
| 43 | Do you regularly take action to reduce your salt intake? | Yes □ (1)<br>No □ (2)<br>Doesn't know □ (3)<br>Refuses □ (88) |
| 44 | What actions do you take to reduce your salt/sodium intake?<br>*(Choose one or more)* | Avoiding/reducing processed foods □ (1)<br>Verifya sodium/salt content of food □ (2)<br>Not adding salt at the table □ (3)<br>Salt substitutes □ (4)<br>Sodium substitutes □ (5)<br>Not adding salt when cooking □ (6)<br>Using spices when cooking □ (7)<br>Avoid eating out □ (8)<br>Other (specify) (9) ________________<br>None □ (10) |

| **During free time** | | |
|---|---|---|
| | | |
| 54 | During your free time, do you practice any intense sport or physical activity that imply a significant acceleration of hearth or breathing rate (running, football) for at least 10 straight minutes? | Yes□<br>(1)<br>No □ (0) |
| 55 | During a typical week, how many days do you have to carry out intense physical activity during your free time? | Number of days |
| 56 | During any of these days, how much time do you spend on these intense physical activities? | Hours: Minutes: |
| 57 | During your free time, do you practice any moderate sport or physical activity that imply a slight acceleration of hearth or breathing rate (walking, biking, volleyball) for at least 10 straight minutes? | Yes□<br>(1)<br>No □ (0) |
| 58 | During any of these days, how much time do you spend on these moderate physical activities? | Number of days |

| | | | |
|---|---|---|---|
| [illegible] | [illegible] moderate physical activities? | | |

| Sedentary lifestyle | | | |
|---|---|---|---|
| 63 | During a typical day, how much time do you spend sitting or lying down? (exclude sleeping) | Hours: | Minutes: |

| Transportation during free time | | |
|---|---|---|
| 60 | Do you walk or use bicycle for at least 10 straight minutes for transportation during your free time? | Yes□ (1) |
| 61 | How many days a week do you walk or use bicycle for at least 10 straight minutes for transportation during your free time? | Number of days |
| 62 | How much time do you spend walking or using bicycle for at least 10 straight minutes for transportation during your free time? | Hours: minutes: |

| PERSONAL MEDICAL HISTORY | | |
|---|---|---|
| Question | | Anser |
| 64 | Have you been diagnosed with heart failure? | Yes □ (1)<br>No □ (0) |
| 65 | Have you been diagnosed with miocardial infarction? | Yes□ (1)<br>No □ (0) |
| 66 | Have you been diagnosed with a "heart condition"? | Yes □ (1)<br>No □ (0)<br>If yes, specify________________ |
| 67 | Have you been diagnosed with brain stroke? | Yes □ (1)<br>No □ (0) |
| 68 | Have you been diagnosed with kidney disease? | Yes □ (1)<br>No □ (0) |
| 69 | Have you been diagnosed with peptic ulcer? | Yes □ (1)<br>No □ (0) |
| 70 | Have you been diagnosed with liver disease? | Yes □ (1)<br>No □ (0) |
| 71 | Have you been diagnosed with cancer or malignant tumor? | Yes □ (1)<br>No □ (0) |

## 3 – Mediciones físicas

| MEASURMENTS | | |
|---|---|---|
| 84 | Blood pressure, measurement 1 | Systolic (mmHg) __ __ __<br>Diastolic (mmHg) __ __ __ |
| 85 | Blood pressure, measurement 2 | Systolic (mmHg) __ __ __ |

| **HYPERTENSION** | | |
|---|---|---|
| **Question** | | **Anser** |
| 72 | Have you ever had your blood pressure measured | Yes (1) No (0) |
| 74 | During the last 12 months? | Yes □ (1) No □ (0) |
| 75 | *Are you currently under any of the following treatments for high blood pressure?* | |
| | Medications taken in the last two weeks | Yes □ (1) No □(0) |
| | Advice to reduce salt intake | Yes □ (1) No □(0) |
| | Advice to lose weight | Yes □ (1) No □(0) |
| | Advice to stop smoking | Yes □ (1) No □(0) |
| | Advice to excercise more | Yes □ (1) No □(0) |
| 76 | Have you ever seen a "healer" due to your high blood pressure? | Yes □ (1) No □(0) |
| 77 | Are you currently taking any herbs or alternative medicine to treat high blood pressure? | Yes □ (1) No □(0) |

| | | |
|---|---|---|
| 86 | Blood pressure, measurement 3 | Systolic (mmHg) __ __ __ |
| | | Diastolic (mmHg) __ __ __ |
| 87 | **Heart rate** | |
| | Measurement 1 | Beats per minute __ __ __ |
| | Measurement 2 | Beats per minute __ __ __ |
| | Measurement 3 | Beats per minute __ __ __ |

| **DIABETES** | | |
|---|---|---|
| 78 | Have you ever had your blood glucose measured | Si □ (1) No □ (0) |
| 79 | Have you ever been told that you have high blood glucose? | Si □ (1) No □ (0) |
| 80 | During the last 12 months? | Si □ (1) No □ (0) |
| 81 | *Are you currently under any of the following treatments for high blood glucose?* | |
| | Medications taken in the last two weeks | Si □ (1) No □ (0) |
| | Advice to reduce salt intake | Si □ (1) No □ (0) |
| | Advice to lose weight | Si □ (1) No □ (0) |
| | Advice to stop smoking | Si □ (1) No □ (0) |
| | Advice to excercise more | Si □ (1) No □ (0) |
| | Have you ever had your blood pressure measured | Si □ (1) No □ (0) |
| 82 | Have you ever seen a "healer" due to your high blood glucose? | Si □ (1) No □ (0) |
| 83 | Are you currently taking any herbs or alternative medicine to treat high blood glucoes? | Si □ (1) No □ (0) |

| **Risky eating behaviours** | | |
|---|---|---|
| **Question** | | **Anser** |
| 88 | Have you ever had fear of gettig fat? | Never □ (1)<br>Almost never □ (2)<br>Sometimes □ (3)<br>Frequently (2 times per week) □ (4)<br>Very frequently (more than 3 times per week) □ (5) |
| 89 | Have you ever eaten more excessively with the feeling that you cannot stop doing it? | Never □ (1)<br>Almost never □ (2)<br>Sometimes □ (3)<br>Frequently (2 times per week) □ (4)<br>Very frequently |

| | | |
|---|---|---|
| | | (more than 3 times per week) □ (5) |
| 90 | Have you ever fasted o used any action to lower your weight like vomiting, using laxatives or excessive excercise? | Never □ (1)<br>Almost never □ (2)<br>Sometimes □ (3)<br>Frequently (2 times per week) □ (4)<br>Very frequently (more than 3 times per week) □ (5) |
| 91 | Have you ever been on a diet to lose weight? | Never □ (1)<br>Almost never □ (2)<br>Sometimes □ (3)<br>Frequently (2 times per week) □ (4)<br>Very frequently (more than 3 times per week) □ (5) |
